# Chronic kidney disease progression rate and predictors in hypertensive patients: A 5-year retrospective follow-up study in Ethiopia

**DOI:** 10.1371/journal.pone.0355483

**Published:** 2026-08-13

**Authors:** Getaneh Atikilt Yemata, Birhanemaskal Malkamu, Fisseha Nigussie Dagnew, Mebratu Libanos, Tewodros Wossen Gemechu, Marelign Tilahun, Wondimnew Desalegn Addis, Mulu Tiruneh

**Affiliations:** 1 Department of Public Health, College of Health Science, Debre Tabor University, Debre Tabor, Ethiopia; 2 Department of Medical Laboratory Sciences, College of Health Sciences, Debre Tabor University, Debre Tabor, Ethiopia; 3 Department of Pharmacy, College of Health Sciences, Debre Tabor University, Debre Tabor, Ethiopia; 4 Department of Internal Medicine, School of Medicine, College of Health Sciences, Debre Tabor University, Debre Tabor, Ethiopia; 5 Department of Epidemiology and Biostatistics, Institute of Public Health, College of Medicine and Health Sciences, University of Gondar, Gondar, Ethiopia; PLOS: Public Library of Science, UNITED STATES OF AMERICA

## Abstract

**Introduction:**

Chronic kidney disease is a major public health concern worldwide. Despite the well-established link between hypertension and chronic kidney disease, early detection and management of chronic kidney disease among hypertension patients in primary healthcare settings remain inadequate. Moreover, studies on the progression and predictors of chronic kidney disease among hypertensive individuals in Ethiopia, particularly in the study area, are limited. Therefore, this study aimed to assess chronic kidney disease progression rate, incidence, and predictors among hypertensive patients in South Gondar Zone hospitals in Ethiopia.

**Methods:**

A retrospective follow-up study was conducted among the medical records of 510 hypertensive patients in South Gondar Zone hospitals, North West Ethiopia, from September 2019 to June 2024, which was selected through simple random sampling technique. Data were collected via a structured checklist with an Android-based Kobo collection toolbox from the medical charts. EPI data version 4.2 and STATA version 17 were used for data entry and analysis. Kaplan‒Meier, log-rank tests, and a Cox proportional hazard regression model were used for data analysis.

**Result:**

Progression to chronic kidney disease occurred at a median of 46 months (IQR:37–55) in hypertension patients. The incidence rate of chronic kidney disease among hypertensive patients was 9.45 (95% CI: 8.07, 11.01) per 1000 person-month observations. Uncontrolled blood pressure (AHR = 1.52, 95% CI: 1.05–2.20), obesity (AHR = 2.56, 95%CI: 1.63–4.01), and high triglyceride levels (AHR = 1.72, 95%CI: 1.03–2.91) were significant predictors of rapid progression of chronic kidney disease among hypertensive patients as compared with their counterparts.

**Conclusion and recommendation:**

Hypertensive patients in this study progressed to chronic kidney disease more rapidly as compared to other studies. This could be explained by obesity, uncontrolled blood pressure, and high triglyceride levels. Routine screening of triglyceride levels and lifestyle modifications that target obesity reduction and impact blood pressure control is recommended.

## Introduction

Chronic kidney disease (CKD) is a condition characterized by a progressive decrease in kidney function over time that results in decreased kidney capacity to excrete water-soluble waste products and adjust water, electrolytes, and acid-base balance [1]. It affects millions of adults, particularly those with diabetes and hypertension [2], ranging from early Glomerular Filtration Rate (GFR) decline to kidney failure, with hypertension as a main contributing factor [3,4].

CKD is a global issue, and it is increasingly common worldwide, affecting 10% of the world’s population (over 85 million people). Moreover, CKD is a growing non communicable disease, more common in women 14% [5]. Although there are different and complex causes of CKD, its early signs are frequently subtle and rare [6]. The World Health Organization (WHO) predicted that CKD would be the fifth most prevalent chronic disease by 2040 [7].

According to the Global Burden of Disease (GBD) study, there are approximately 19 million incident cases, over 400,000 deaths, and over 41 million Disability Adjusted Life Years (DALYs) attributed to CKD worldwide, indicating an increasing trend in the global burden of CKD. Notably, high systolic blood pressure, particularly in poorer nations, is a major drivers of CKD related DALYs and mortality [8–10].

Incidence rates of CKD vary significantly by region: Europe(Denmark) reports approximately 49 cases of stage 3–5 CKD per 10,000 person years [11], and US reports 64 cases of CKD per1, 000 person years among diabetic adults [12]. A global estimate shows a dramatic increase in CKD incidence of 25.3 per 1,000 person years in high risk population and 6.3 per 1,000 person years in the general population [13]. In Africa a systematic review pooled prevalence of CKD indicates 18% in the general population and 32.3% among high-risk individuals (those with diabetes and hypertension) [14,15], but incidence data remains limited. Collectively these findings underscores a rising and regionally heterogeneous global CKD burden, with low income settings facing distinct risk factor challenges [16].

CKD progression was considered an indolent process, typically unfolding over many years or even decades. However, emerging evidences challenges this notion by demonstrating that a subset of patients experience rapid renal function decline. In particular, data from recent studies show a progression rate 50% reduction in e GFR over three years, and a 57% loss of kidney function within 32 months [17,18]. Nevertheless, these data originate exclusively from developed countries, and there remains a lack of conclusive and updated evidence regarding CKD progression in low income countries. The progression of chronic kidney disease (CKD) is influenced by a range of interconnected predictors, including metabolic factors, lifestyle behaviours, genetic predisposition, and the presence of key comorbidities such as diabetes mellitus and cardiovascular disease [19,20].

Chronic kidney disease has far-reaching societal, economic, and public health consequences because of its progressive nature, high treatment costs, and impact on quality of life. Chronic kidney disease significantly impacts global health, contributing to increased disability and mortality. Despite advancements in healthcare, the burden of CKD continues to rise, underscoring the need for enhanced prevention, early detection, and management strategies to mitigate its global impact [8,21].

The ongoing epidemiologic shift from communicable to Non-Communicable Diseases (NCDs), especially CKD and CVD, strongly contributed by hypertension, double the challenge of low and middle income countries healthcare system [22]. These countries are experiencing an epidemic of CKD of unknown origin, and inadequate access to early diagnosis and dialysis worsens outcomes [23].

Ethiopia, face a triple burden of infectious disease, injuries, and rising incidence of NCD including CKD, all worsened by poor access to early diagnosis and have devastating impacts on the nation’s health sector and economy [24]. According to the WHO NCD report in Ethiopia 39% of deaths are attributable to NCDs, including CKD and hypertension [25]. According to a study conducted in Ethiopia, the prevalence of CKD in the general adult population ranged from 14.6% to 19.1%. However data on new cases, and progression rate with contextualized factors are limited [26,27].

Hypertension is a leading cause of CKD, yet many patients remain undiagnosed until the disease has advanced, leading to increased morbidity, higher hospitalization rates, and costly treatments such as dialysis and kidney transplants [28,29]. Despite the well-established link between hypertension and CKD, studies on the incidence, progression rate, and early detection in primary healthcare settings remain limited [30,31].

Therefore, this study aimed to investigate the incidence, progression rate, and predictors of CKD among hypertensive patients in primary health care settings of South Gondar Zone hospitals. By addressing these gaps, this study can contribute to reducing CKD-related complications, improving patient outcomes, and alleviating the healthcare burden.

## Methods and materials

### Study design and period

We carried out an institution-based retrospective follow-up study to determine the incidence, and progression time of chronic kidney disease, as well as its predictors, among hypertension patients who received follow-up care in selected hospitals between September 2019 and June 2024. Medical records of the study participants with a diagnosis of hypertension and chronic care follow-up at the chosen hospitals were identified by the investigator. Subsequently, the researcher looked retrospectively at the patients’ case status at the beginning of the study and only hypertension cases free of kidney disease were selected to track over time to the desired outcome, such as an event or censoring. At the beginning of the study, the study population’s exposure status or potential confounders were identified.

### Study setting

The study was conducted in selected hospitals in the South Gondar Zone, North-Western Ethiopia. According to the Central Statistical Agency population projection of Ethiopia, the South Gondar zone has a population of 2,631,566; of these, 1,331,888 are males, and 1,299,678 are females. There are 21 woredas and 401 kebeles in the South Gondar Zone. In addition, in the South Gondar zone, one comprehensive specialized hospital and nine primary hospitals were found. A total of ten hospitals are located in South Gondar Zone district of Northwest Ethiopia. These hospitals a provide inpatient and outpatient services, including hypertension care, with different levels of healthcare workers. From these, three hospitals named Debre Tabor Comprehensive Specialized Hospital (DCSH), Nefas Mewucha Primary Hospital (NMPH), and Addis Zemen Primary Hospital (AZPH) were selected using a lottery method, and our study was conducted in these three hospitals.

### Population

All medical charts of hypertensive patients receiving treatment and follow-up at South Gondar Zone hospitals composed our source population, whereas all adult hypertensive patients who received follow-up during the last five years at the selected hospitals in South Gondar Zone composed the study population.

Eligibility**:** All adult hypertensive patients who were followed up for at least 6 months and aged above 18 years were included in the study. However, pregnant women, patients with chronic kidney disease at the beginning of the study, and patients with incomplete medical records on outcome variables were excluded.

### Sample size determination and sampling technique

The required sample size was determined via Epi Info software for cohort study sample size calculation with the assumptions of a 95% confidence level, 80% power, and a ratio of nonexposed to exposed samples of 1:1. In addition, the effects of uncontrolled blood pressure, dyslipidaemia, and baseline blood pressure were measured to calculate the required sample size. Among these factors, baseline blood pressure was chosen as an exposure variable because it provided the maximum sample size compared with the other variables. Since the proportion of exposure among the outcomes was 26.4%, the hazard ratio was 1.72, [32]. After adding 10% of the missing data, the sample size was determined to be 528.

Three of the 10 hospitals in the North-Western Ethiopia, South Gondar zone district that provide hypertension care services were chosen for this study via a simple random selection procedure. The total sample size was proportionally allocated for the selected hospitals.

Based on the follow-up registry of hypertensive patients, 1420 patients at Debre Tabor Comprehensive Specialized Hospital, 488 patients at Nefas Mewucha Primary Hospital, and 592 patients at Addis Zemen primary hospitals underwent follow-up during the study period. The total sample size was divided based on this proportion, resulting in final sample sizes of 300 from Debre Tabor Comprehensive Specialized Hospital, 103 from Nefas Mewucha Primary Hospital, and 125 from Addis Zemen primary hospitals. A sampling frame was prepared based on their card number (the list of hypertensive patients enrolled from September 2019 to June 2024)), and simple random sampling with a computer-generated random sample was used to select each participant.

### Study variables

**Dependent variables**: Progression time to hypertensive CKD and Incidence of hypertensive CKD.

**Independent variables:** Sex, age, residence, marital status, occupation, education, body mass index (BMI), blood pressure control, low-density lipoprotein (LDL), triglycerides, baseline blood pressure level, diabetes mellitus comorbidity, hypertensive drug usage, and non-hypertensive drugs usage.

### Measurement

The progression time was the follow-up time in months between the patient’s hypertension diagnosis and the occurrence of the outcome (event or censored). Hypertensive patients who acquired chronic kidney disease were considered as outcome of interests or events. Patients with no documented evidence of chronic kidney disease, unclear diagnoses, and transfers to other medical facilities without knowledge of the results were considered censored. Survival status is the outcome status of hypertensive patients at the end of the follow-up for each participant, either having an event or being censored.

Hypertension is defined as a previous diagnosis of and ongoing treatment for hypertension or a record of sustained blood pressure ≥140/90 mm Hg on two or more occasions [33].

Hypertensive chronic kidney disease was defined as a history of hypertension accompanied by at least one of the following criteria, present for ≥3 months and diagnosed by a physician and documented on the medical chart: 1. Reduced kidney function-estimated Glomerular Filtration Rate (eGFR) < 60 mL/min/1.73 m²: based on the Cockcroft‒Gault equation. 2. The markers of kidney damage measured through protein urea by dipstick testing (proteinuria ≥1**+**) on at least two occasions [34].

Estimated GFR is a test used to evaluate how well the kidneys are functioning and estimates how much blood the kidneys filter throughout a specific time frame. The eGFR can be calculated using widely available Cockcroft‒Gault formula (normalized for Body Surface Area [BSA]) as follows: (140 minus years of age) × weight (kg) × (0.86, if female) × 1.73/72 × serum creatinine (mg/dL) × BSA (m^2^). BSA is calculated using Mosteller formula as BSA (m^2^) = (√Height ×Weight) divided by 60, where weight is measured in kilogram and height in centimetre [35].

According to the International Classification of Disease Tenth Revision (ICD-10) version 2019, designed by the WHO, hypertensive chronic kidney disease stage 1 to stage 4 or unspecified chronic kidney disease is coded under the range of the circulatory system as I12.9 [36].

Blood pressure was controlled as follows: BP < 140/90 mmHg at the last follow-up via a digital sphygmomanometer for adult hypertensive clients without diabetes mellitus and chronic kidney disease and blood pressure <130/80 mmHg via a digital sphygmomanometer for adult hypertensive clients with diabetes mellitus and chronic kidney disease at the last follow-up record [37].

According to the American Heart Association and National Cholesterol Education Program, lipid biomarkers, which consider clinical significance, are defined as LDL cholesterol levels: optimal: < 130 mg/dL (2.6 mmol/L); borderline high: 130–159 mg/dL (3.4–4.1 mmol/L); high: > 160 mg/dL (4.1–4.9 mmol/L); triglyceride, normal: < 150 mg/dL (<1.7 mmol/L); borderline high: 150–199 mg/dL (1.7–2.2 mmol/L); and high: > 200 mg/dL (2.3–5.6 mmol/L) [38–40].

Body mass index (BMI) was classified as normal if it was less than 24.9 kg/m2, overweight if it was between 25 and 29.9 kg/m2, and obese if it was greater than 30 kg/m2 [41]. Proteinuria was defined as a dipstick result of +1 (≥30 mg/dL) or higher in at least two separate tests [42].

### Data collection method

Chart review using a structured checklist which was developed after a review of relevant pieces of literature and other materials that can address the objectives of the study was used to collect the data [43–47]. Sociodemographic, clinical, laboratory, and dates of important time points (e.g., diagnostic date of hypertension, date of outcome occurrence, i.e., event or censored), and outcome data were the questionnaire’s components. The diagnosis of hypertensive chronic kidney disease was confirmed by blood testing, urine testing, and renal ultrasound. The baseline level of clinical and lab-based variables was taken. Data were extracted from medical records of hypertension patients who were followed at the selected hospitals between September 2019 and June 2024. An electronic-based data collection tool, i.e., the Android-based Kobo collection toolbox was used to retrieve the data between September and October of 2024. The data were collected by six trained health professionals (BSc nursing) who work on the study site. One supervisor per site (General practitioner)) was recruited to supervise the data collection process.

### Data quality assurance

To maintain the quality of the data, a comprehensive search and review of relevant literature were applied to prepare a standardized checklist. Health experts with experience in the research field checked the tool’s face and content validity and compliance with the study’s objective. The study’s objectives, the data-gathering process, the nature of the tool, how to use the Kobo collection tools in mobile applications, and other pertinent tasks were addressed in two days of training for the supervisor and data collectors.

A pre-test was conducted on 5% of the sample at Debre Tabor Comprehensive Specialized Hospital, and necessary modifications were made; these medical records were excluded from the final analysis. Throughout the data collection period, the procedure was closely monitored every day. The Kobo collect toolbox app controlled the data completeness and accuracy, and further, the supervisors checked in the daily site inspections. In addition, the investigator gave feedback after overviewing the data in the server and reviewed the data before it was exported to the other software for analysis. Before analysis, the data were examined for completeness and internal consistency.

### Data analysis

The data were entered into Epi Data version 4.1 and exported to STATA version 17 for analysis. The variables occupation, smoking, HDL, and total cholesterol were removed because the data were incomplete on around 60–70% of study subjects, making reliable imputation impractical. Complete case analysis was performed and the list-wise deletion method was applied for the missing data due to the assumption that missing data is Missing Completely at Random (MCAR) and small in proportion (<5%) which was 3.4%. For variables with moderate missingness like marital status, LDL and triglycerides, multiple imputation method was applied to handle missing values. This method creates several imputed datasets by predicting missing values based on the observed data and relevant covariates, and then combine the results across datasets to produce unbiased estimates.

The incidence rate was determined by dividing the total number of events by the total person-months at risk. Median with interquartile range (IQR) was used to report the progression time for patients who developed chronic kidney disease. Kaplan‒Meier method was applied to estimate the probability of hypertensive patients to develop the event at different time, mean and median progression time. Moreover, Log-rank tests were used to compare progression times across different predictor variable categories. For pairwise or multiple comparisons, a post hoc log-rank test was used to assess the significance of survival differences between covariate categories. The sociodemographic, laboratory, and clinical characteristics of the hypertensive patients were summarized via descriptive statistics and presented thorough frequency and percentage.

A bi-variable Cox proportional hazards regression model was fitted for each explanatory variable and those variables with a p-value <0.25 in the bivariate analysis were fitted to the multivariable Cox proportional hazards regression model. The significant predictors were declared at a P value of less than 5%. The Cox PH regression model assumption was checked via the Cox-Snell residuals test, Schoenfeld residual test or a global test, and multi-collinearity. The regression model was fitted and assessed for adequacy using Schoenfeld residuals, the Cox-Snell residual test, and multi-collinearity diagnostics. The model was found to be adequate. The adjusted hazard ratio (AHR) with 95% confidence interval (CI) and P value were used to measure the association strength and identify statistically significant predictors [48].

### Ethical consideration

The study protocol was developed by the study team and reviewed and approved by the Debre Tabor University Institutional Ethical Review Committee (DTUEIRC). Following authorization by the research ethics committee, the hospitals were informed about the study through a support letter. Data security and participants’ confidentiality were maintained at all levels of data management. In general, this research adheres to the Helsinki Declaration. All methods were performed following the relevant guidelines and regulations.

## Results

The medical records of 528 hypertensive patients who received follow-up care from September 2019 to June 2024 at the three selected hospitals in the South Gondar Zone were chosen. However, 18 medical charts were excluded from the study due to the incompleteness of pertinent research data. Overall, data were analysed only on 96.6% (510) of the selected medical charts of hypertensive patients.

### Baseline characteristics of hypertensive patients who were followed up in South Gondar Zone hospitals

The majority of the study participants (304, 59.6%) were 60 years of age or older, and approximately 379 (74.3%) were urban residents. Female participants (307, 60.2%) were in higher proportion compared with males (203, 39.8%), reflects the health care seeking behaviour in this setting, where women are more likely to attend regular follow-up visits for hypertension care. Additionally, close to one-third of the hypertensive patients (169, 33.2%) were obese. Nearly 211 (41.4%) of the study participants had baseline blood pressure greater than 160/100 mmHg, and more than half of them (296, 58.1%)) controlled their hypertension. However, only 87 (17.1%) of the participants had diabetes mellitus comorbidities, and 108 (21.2%) of the patients had a family history of kidney disease (Table 1).

**Table 1:** Baseline sociodemographic and clinical characteristics of hypertensive patients who underwent follow-up at South Gondar Zone hospitals.

Variables	Category	Hypertensive Chronic Kidney Disease		Total: n (%)
		Censored: n (%)	Event: n (%)	
Age	18-39	55(77.5)	16(22.5)	71(13.9)
	40-59	104(77.0)	31(23.0)	135(26.5)
	>=60	185(60.9)	119(39.1)	304(59.6)
Sex	Male	129(63.6)	74(36.4)	203(39.8)
	Female	215(70.0)	92(33.0)	307(60.2)
Residence	Urban	254(67.0)	125(33.0)	379(74.3)
	Rural	90(68.7)	41(31.3)	131(25.7)
Marital status	Single	35(59.3)	24(40.7)	59(11.8)
	Married	182(71.9)	71(28.1)	253(50.6)
	Widowed& divorced	119(63.3)	69(36.7)	188(37.6)
BMI	Normal weight	153(81.4)	35(18.6)	188(36.8)
	Overweight	99(64.7)	54(35.3)	153(30.0)
	Obesity	92(54.4)	77(45.6)	169(33.2)
Duration of hypertension	New onset	75(80.6)	18(19.4)	93(18.3)
	Short term(<1 year)	99(68.8)	45(31.2)	144(28.2)
	Intermediate(1–3 year)	81(63.8)	46(36.2)	127(24.9)
	Long-term (> 3 years)	89(60.9)	57(39)	146(28.6)
Hypertension control	Uncontrolled	108(50.5)	106(49.5)	214(41.9)
	Controlled	236(79.7)	60(20.3)	296(58.1)
Baseline blood pressure	120/80–139/89 mmHg	124(78.0)	35(22.0)	159(31.2)
	140/90–159/99 mmHg	95(67.8)	45(32.2)	140(27.5)
	>160/100 mmHg	125(59.2)	86(40.7)	211(41.4)
DM comorbidity	Yes	44(50.6)	43(49.4)	87(17.1)
	No	300(70.9)	123(29.1)	423(82.9)
Family history of kidney disease	Yes	49(45.4)	59(54.6)	108(21.2)
	No	295(73.4)	107(26.6)	402(78.8)

### Laboratory findings and drug use of hypertensive patients receiving follow-up care in South Gondar Zone hospitals

Approximately one-third of hypertensive patients, (155; 30.4%) and (146; 28.6%), had high levels of low-density lipoprotein cholesterol and triglycerides, respectively. Moreover, ACE inhibitors and diuretic drugs were the commonly used medications in which more than four hundred (402; 78.8%), (425; 82.9%) of the study participants had taken ACE inhibitor and diuretic drugs respectively. Besides many (203; 39.8%) study participants used non-anti-hypertensive drugs (Table 2).

**Table 2:** Baseline lipid biomarkers of hypertensive patients who were followed up in South Gondar Zone hospitals.

Variables	Category	Hypertensive CKD		Total: n (%)
		Censored: n (%)	Event: n (%)	
LDL	High level	78(50.3)	77(49.7)	155(30.4)
	Borderline high	89(64.0)	50(36.0)	139(27.3)
	Optimal level	177(81.9)	39(18.1)	216(42.3)
Triglyceride	High level	69(47.3)	77(52.7)	146(28.6)
	Borderline high	129(70.9)	53(29.1)	182(35.7)
	Normal	146(80.2)	36(19.8)	182(35.7)
ACE inhibitor	Yes	295(73.4)	107(26.6)	402(78.8)
	No	49(45.4)	59(54.6)	108(21.2)
Beta-blocker drug use	Yes	101(77.7)	29(22.3)	130(25.5)
	No	243(64.0)	137(36.0)	380(74.5)
Diuretics drug use	Yes	300(70.9)	123(29.1)	425(82.9)
	No	44(50.6)	43(49.4)	87(17.1)
Calcium channel blocker drug use	Yes	136(68.3)	63(31.7)	199(39)
	No	208(66.9)	103(33.1)	311(60.9)
Vasodilator drug use	Yes	73(76.8)	22(23.2)	95(18.6)
	No	271(65.3)	144(34.7)	415(81.4)
Non-Anti-Hypertensive drug use	Yes	129(63.6)	74(36.5)	203(39.8)
	No	215(70.0)	92(30.0)	307(60.2)

### Incidence rate and progression time to CKD among hypertensive patients undergoing follow-up in South Gondar Zone Hospitals

A total of 510 study subjects were followed and included in the analysis. Approximately 344 (67.45%) observations were censored, and 166 (32.55%) observations developed an event (CKD) with a median duration of 46 months with an IQR of 37–55 months. During the follow-up period, a total of 17,554 person‒month risks were observed, with minimum and maximum follow‒up times of 12 and 60 months, respectively. The overall incidence rate of chronic kidney disease was 9.45 (95% CI: 8.07, 11.01) per 1000 person-month observations.

We found that approximately 945 hypertensive patients out of 100,000 experienced hypertensive chronic kidney disease within a single month. We predicted that approximately 50% of hypertensive patients progressed to chronic kidney disease at 46 months. In addition, we anticipated that 25% and 75% of hypertensive patients wait to progress to CKD only 37 and 55 months respectively.

### Nonparametric analysis of survival data

**Fig 1:**
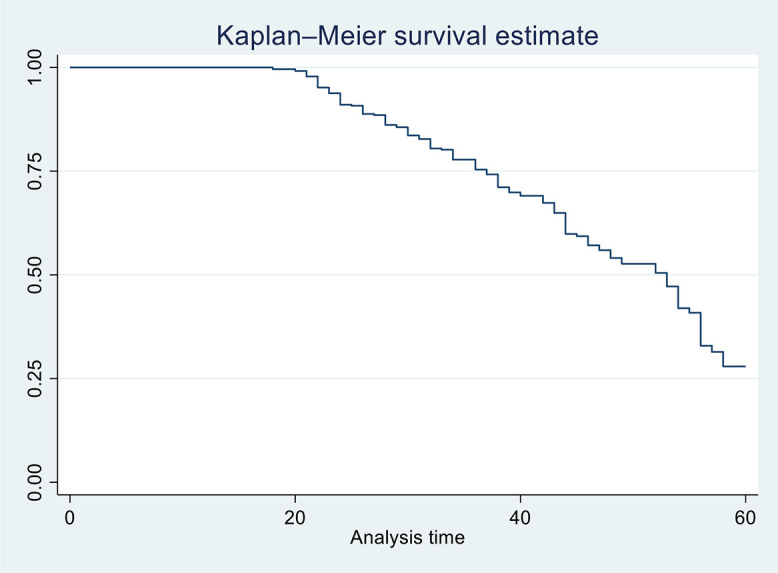
Overall Kaplan –Meier survival estimates.

A Kaplan–Meier estimation technique was used to estimate progression time. The overall graph of the Kaplan–Meier survival function revealed that the value decreased rapidly from 20 months to 55 months, indicating that most patients developed CKD during this time (Fig 1).

**Fig 2:**
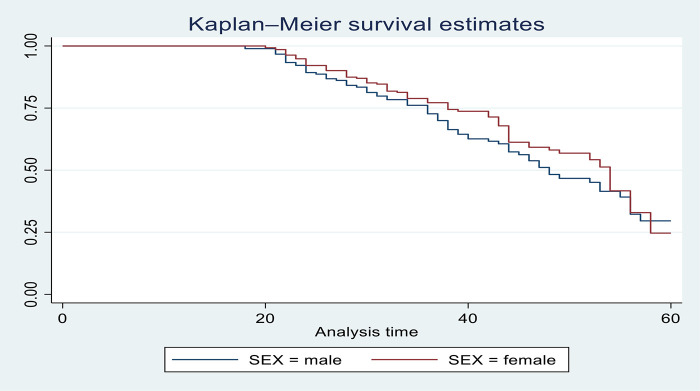
Separate Kaplan–Meier survival function curve estimates by sex.

**Fig 3:**
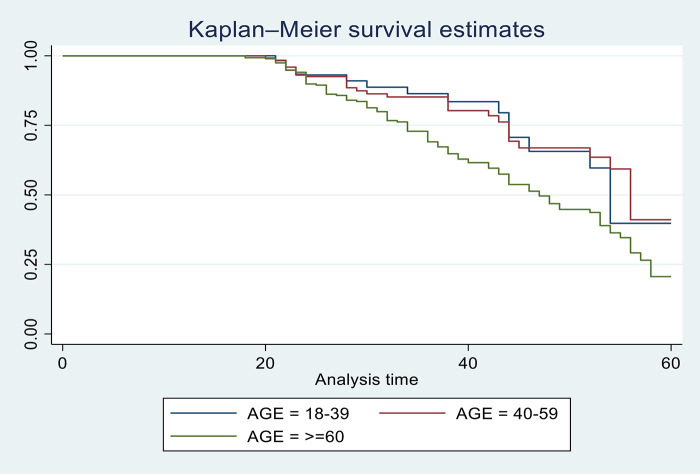
Separate Kaplan–Meier survival function curve estimates by age.

**Fig 4:**
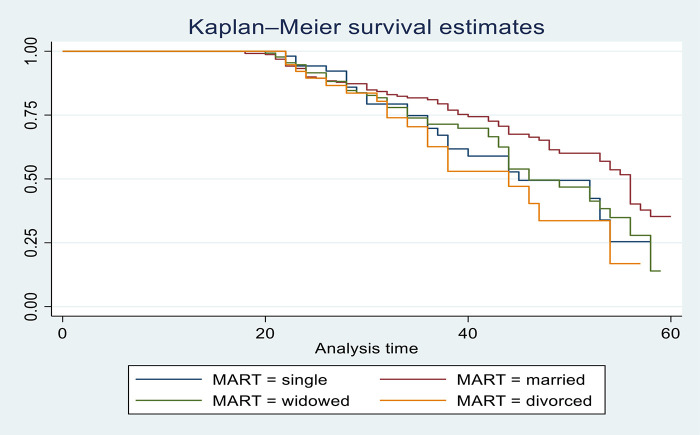
Separate Kaplan–Meier survival function curve estimates by marital status.

**Fig 5:**
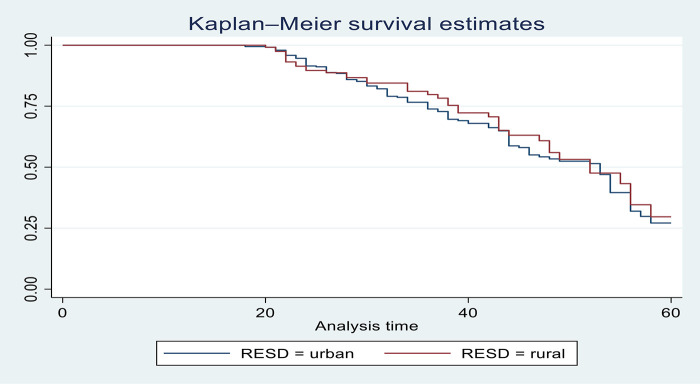
Separate Kaplan–Meier survival function curve estimates by residence.

**Fig 6:**
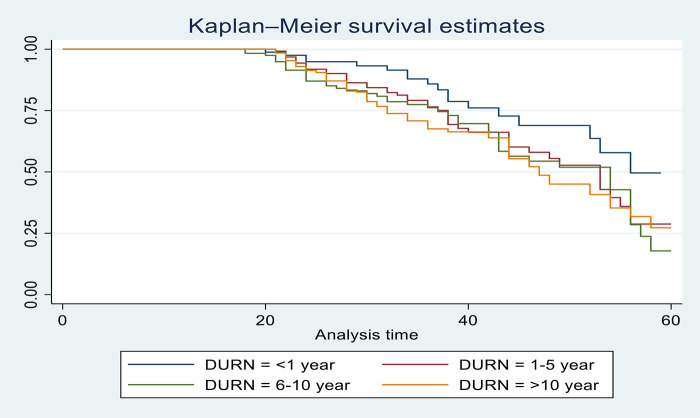
Separate Kaplan–Meier survival function curve estimates by hypertension duration.

**Fig 7:**
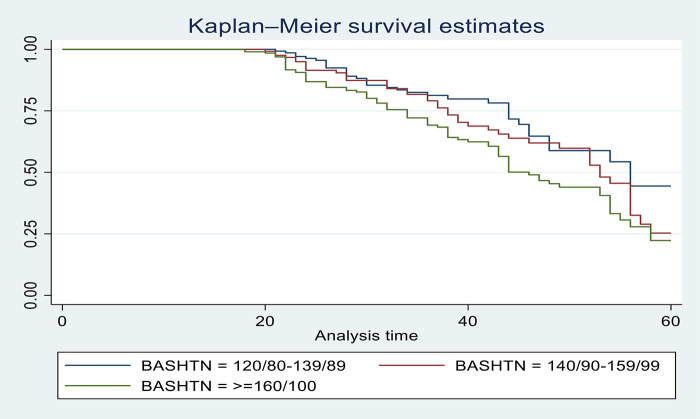
Separate Kaplan–Meier survival function curve estimates by baseline blood pressure.

**Fig 8:**
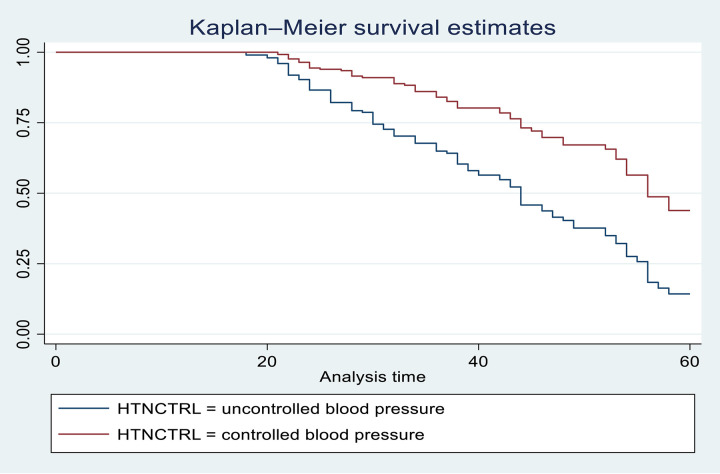
Separate Kaplan–Meier survival function curve estimates by hypertension control.

**Fig 9:**
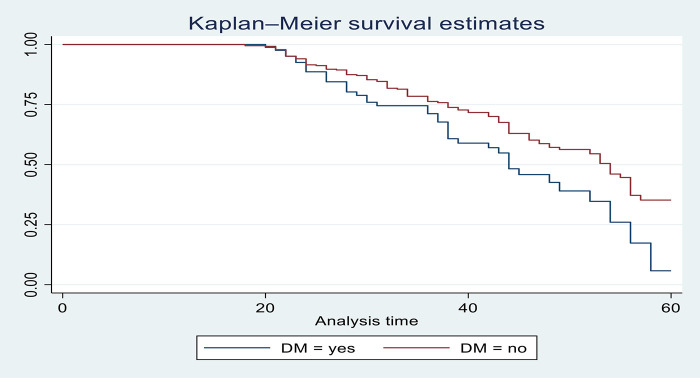
Separate Kaplan–Meier survival function curve estimates by DM.

**Fig 10:**
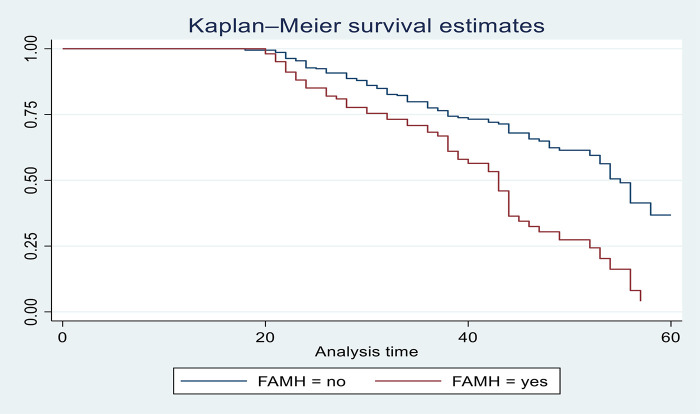
Separate Kaplan–Meier survival function curve estimates by family history of Kidney Disease.

**Fig 11:**
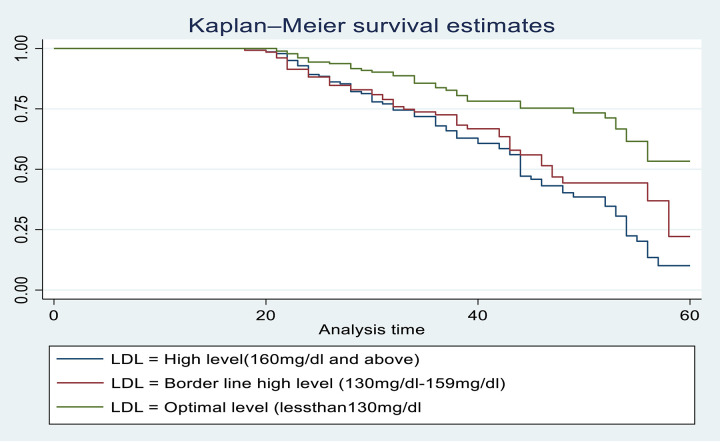
Separate Kaplan–Meier survival function curve estimates by LDL.

**Fig 12:**
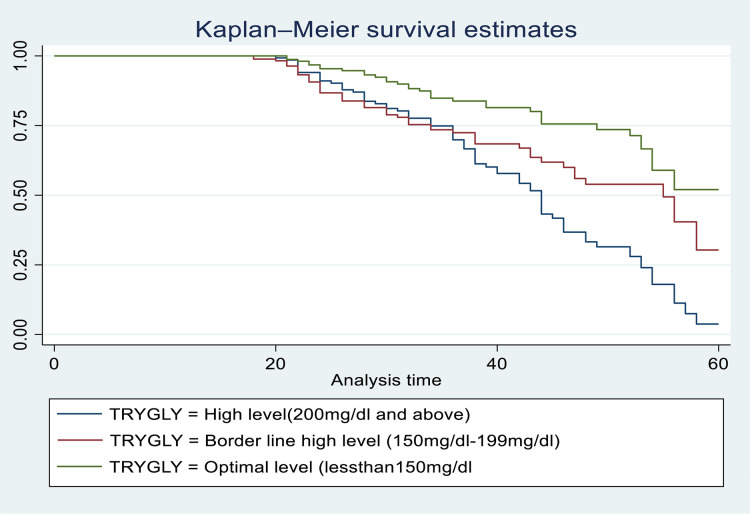
Separate Kaplan–Meier survival function curve estimates by triglyceride.

**Fig 13:**
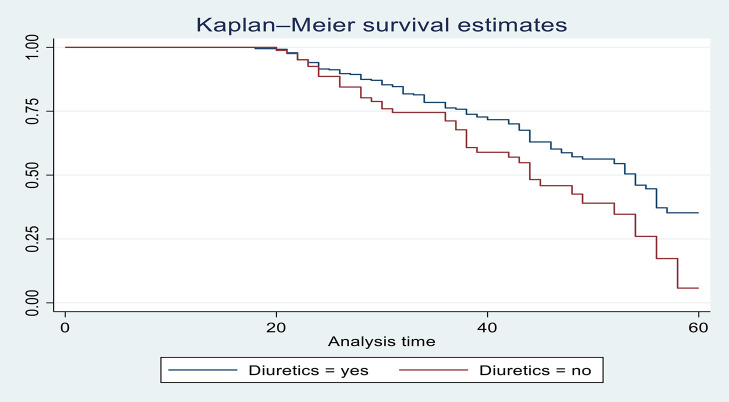
Separate Kaplan–Meier survival function curve estimates by diuretics.

**Fig 14:**
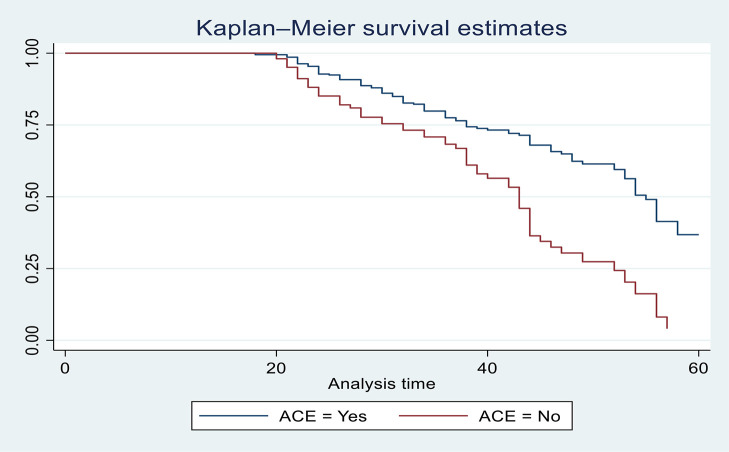
Separate Kaplan–Meier survival function curve estimates by ACE.

**Fig 15:**
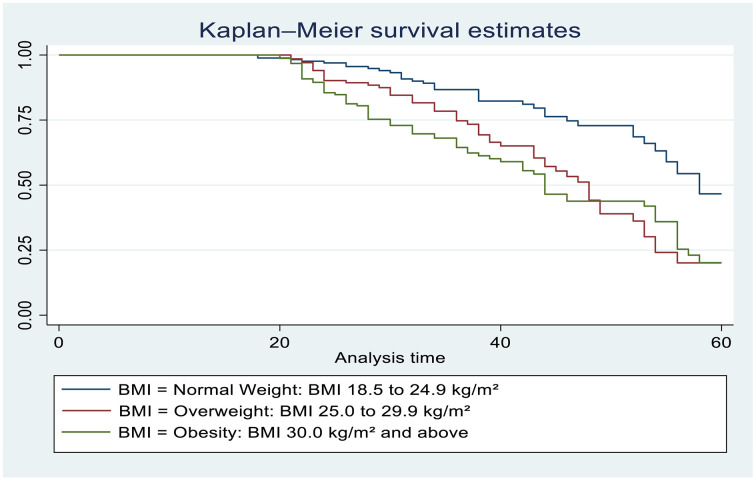
Separate Kaplan–Meier survival function curve estimates by BMI.

A separate Kaplan–Meier survival function curve was constructed to estimate the survival time based on different covariates to determine the difference in the incidence rate of chronic kidney disease between categories of individual covariates. From the Kaplan–Meier survival curve of individual covariates, there was no difference in the incidence rate of CKD among males and females, those residing in urban and rural areas, marital status, duration of hypertension, baseline hypertension, and LDL level. However, there was a difference in the survival probability/incidence rate of CKD for the covariates of older age, diabetes mellitus comorbidity, obesity, family history, and high triglyceride level compared with those of their counterparts (Figs 2–15).

The log-rank test was computed to determine the significance of survival differences at the 5% significance level. Accordingly, there was a significant difference in CKD development concerning age category, DM comorbidity, BMI, LDL, baseline BP, hypertension control status, and triglyceride level (Table 3).

**Table 3:** Log-rank test results for the significant categorical variables of CKD among hypertensive patients who received follow-up care at South Gondar Zone hospitals.

Variables	Chi-square	Df	P value
Age	11.57	2	0.003
Sex	2.65	1	0.1034
Marital status	8.67	2	0.034
Residence	.42	1	.5177
Hypertension control	32.96	1	0.001
Duration of HTN	6.29	2	0.098
Baseline BP	9.45	2	0.008
DM comorbidity	7.86	1	0.005
Family history of CVD	26.53	1	.001
BMI	24.25	2	.001
LDL	28.29	2	0.001
Non-Anti-HTN Drugs	1.18	1	0.27
Triglyceride	61.38	2	.001

### Model selection and adequacy

The Cox proportional hazard regression model assumption was checked via the Schoenfield residual test or global test, Cox-Snell residuals, and multi-collinearity. The hypothesis that the hazard ratios are constant over time was tested via a global test. The results of the global test were statistically insignificant; the p-value = 0.424, which was greater than the tabulated level of significance (0.05). Therefore, the Cox PH model was suitable for these data, and the assumption of a constant hazard ratio over time was satisfied. In addition, the mean variation inflation factor (VIF) for each covariate was less than two, and the mean VIF was 1.2, which indicates no multi-collinearity.

### Predictors of survival time to develop chronic kidney disease

Covariates that had a P value ≤0.25 in the bi-variable Cox regression analysis were selected for multivariable Cox regression analysis. Age category, marital status, blood pressure control status, diabetes mellitus comorbidity, body mass index, low-index lipoprotein cholesterol level, triglyceride level, and non-hypertensive drug use were selected for multivariate Cox regression analysis. Finally, three of the predictors (body mass index, blood pressure control status, and triglyceride level) were found to have a statistically significant association with the early development of hypertensive chronic kidney disease via multivariate Cox proportional regression analysis.

Study subjects who were obese were 2.56 times (AHR = 2.56, 95% CI: 1.63--4.01) more likely to progressed to hypertensive chronic kidney disease than their counterparts. Overweight also increased the risk of hypertensive chronic kidney disease by 1.94 times (AHR = 1.94, 95% CI: 1.21–3.12). Hypertensive patients with uncontrolled blood pressure were 1.52 times more likely to progressed to chronic kidney disease (AHR = 1.52, 95% CI: 1.05–2.20). In addition, the number of hypertensive patients with high triglyceride levels was 2.54 (AHR = 2.54, 95% CI: 1.67, 3.85) times greater than that of their counterparts to progressed to hypertensive chronic kidney disease (Table 4).

**Table 4:** Cox proportional hazards regression analysis of time to chronic kidney disease development among hypertensive patients who underwent follow-up at South Gondar Zone Hospitals.

Variables	Category	Chronic Kidney disease Event censored		CHR,95%CI	P Value	AHR,95%CI	P Value
Age	18-39	55(77.5)	16(22.5)			1	
	40-59	104(77.0)	31(23.0)	0.99(0.54-1.82)	0.98	1.35 (.66-2.75)	0.41
	>=60	185(60.9)	119(39.1)	1.76(1.04-2.96)	0.03	1.45 (.81-2.62)	0.20
Marital status	Single	35(59.3)	24(40.7)			1	
	Married	182(71.9)	71(28.1)	0.66(0.42-1.06)	0.09	0.72 (.41-1.24)	0.242
	Widowed & Divorced	119(63.3)	69(36.7)	1.27(0.69-2.32)	0.43	1.68(0.80-3.51)	0.16
Hypertension control status	Uncontrolled	108(50.5)	106(49.5)	2.41(1.76-3.32)	0.001	1.52 (1.05-2.20)	**0.03**
	Controlled	236(79.7)	60(20.3)	1		1	
Duration of hypertension	New onset	75(80.6)	18(19.4)			1	
	Short term(<1 year)	99(68.8)	45(31.2)	1.62(0.93-2.79)	0.08	1.26 (.69-2.30)	0.444
	Intermediate(1–3 year)	81(63.8)	46(36.2)	1.78(1.03-3.08)	0.03	1.37 (.75-2.49)	0.302
	Long-term (> 3 years)	89(60.9)	57(39)	1.89(1.11-3.22)	0.01	1.38 (.77-2.48)	0.272
Baseline BP	120/80-139/89	124(78.0)	35(22.0)				
	140/90-159/99	95(67.8)	45(32.2)	1.32(0.84-2.05)	0.21	1.14(.70-1.85)	0.588
	>=160/100	125(59.2)	86(40.7)	1.78(1.20-2.64)	0.004	1.32 (.83-2.09)	0.226
DM comorbidity	Yes	44(50.6)	43(49.4)	1.62(1.14-2.29)	0.006	1.22 (0.81-1.84)	0.33
	No	300(70.9)	123(29.1)				
LDL level	High level	78(50.3)	77(49.7)	2.14(1.41-3.25)	0.001	1.08 (.62-1.86)	0.28
	Borderline high	89(64.0)	50(36.0)	0.85(0.53-1.37)	0.52	1.20 (.69-2.09)	0.50
	Normal	177(81.9)	39(18.1)			1	
BMI	Normal weight	153(81.4)	35(18.6)			1	
	Overweight	99(64.7)	54(35.3)	2.20(1.43-3.37)	0.001	1.94(1.21- 3.12)	**0.006**
	Obesity	92(54.4)	77(45.6)	2.55(1.71-3.81)	0.001	2.56(1.63-4.01)	**0.001**
Triglyceride	High level	69(47.3)	77(52.7)	2.99(2.01-4.46)	0.001	1.25(0.72-2.16)	**0.04**
	Borderline high	129(70.9)	53(29.1)	1.91(1.25-2.93)	0.003	1	0.42
	Normal	146(80.2)	36(19.8)				
Non-HTN drugs	Yes	129(63.6)	74(36.5)	1.81(0.86-1.60)	0.28	1.34 (0.86-2.11)	0.190
	No	215(70.0)	92(30.0)				

## Discussion

This study aimed to determine the incidence, progression time, and predictors of chronic kidney disease among hypertensive patients undergoing follow-up in South Gondar Zone Hospitals.

Our study revealed that 50% of hypertensive patients progressed to CKD at approximately four years (46 months) with an IQR of 37–55 months. The progression rate in this finding is more rapid than a study conducted in some of the African countries like Ghana [49], and West Africa [50]. Surprisingly, this Ethiopian finding is in line with a study conducted in South Africa [51], China [52], Australia [53], and the United States of America (Maryland) [54]. These studies, including ours, revealed that hypertensive patients, especially those with poorly controlled BP (>140/90 mmHg), have a 25–75% risk of CKD within 3–5 years, and 50% of them are at risk of CKD at approximately four years. This could be because kidney function in hypertension patients may decline at a rate of 3–5 mL/min/1.73 m² per year, making CKD evident at 3 years in high-risk individuals [55]. This finding implied that individuals with hypertension had a more rapid decline in the estimated glomerular filtration rate or accelerated kidney function decline. Therefore, early and urgent screening and intervention are essential to prevent the development of CKD and its adverse outcomes. In addition to the biological mechanism, this rapid progression in Ethiopia would be attributed to high prevalence of uncontrolled hypertension, shortage of specialists, limited health care facility for screening, lack of early medical intervention, and competing health priorities from infectious disease and maternal health [56].

Concerning the incidence rate of hypertensive chronic kidney disease, approximately 945 out of 100,000 hypertensive individuals developed hypertensive heart disease within one month. These findings are consistent with studies in Tehran, Iran. [57], Korea [31], and Poland [58]; a review study in Ethiopia [59]; and the selection of referral hospitals in the Amhara region, Ethiopia [60]. However, the incidence rate of hypertensive chronic kidney disease is lower than that reported in studies conducted in Northcentral Ethiopia. [61], Tigray Teaching Hospitals, Tigray, Ethiopia [62], Jimma referral hospitals, and Jimma, Ethiopia [63]. The observed discrepancies may be because our study investigated only new cases of CKD, whereas a study in Tigray, Jimma, and North Central Ethiopia assessed the prevalence of CKD without differentiating between new-onset and pre-existing disease. In addition, the level of health care, quality of medical service, sample size, and lifestyle modification practices may have contributed to the discrepancy.

The current study revealed that hypertensive patients with obesity are at approximately three times greater risk of developing chronic kidney disease than patients with normal body weight. Existing evidence supports the present study’s findings such as a study in Tehran Iran (Tehran glucose and lipid study) [57], mainland China [64], ST. Paul Hospital Addis Ababa Ethiopia [65], and North Central Ethiopia [61].

Obesity exacerbates hypertension and accelerates the progression of chronic kidney disease through interrelated mechanisms, including metabolic disorders, production of inflammatory cytokines, and direct kidney damage. For example, obesity induces a chronic low grade inflammatory state characterized by increased production of pro-inflammatory cytokines such as tumor necrosis factor-alpha, interlukin-6 and C- reactive protein. These inflammatory mediators contribute to endothelial dysfunction, glomerular hyper filtration, forcing the kidneys to work harder to filter waste, which can result subsequent kidney damage and kidney function decline, thereby accelerating the progression to CKD in hypertensive patients with obesity. Additionally, obesity triggers RAAS over activation, causing renal fibrosis, vasoconstriction, and salt retention, further worsening hypertension and accelerating CKD progression [66,67].

This study revealed that in hypertensive patients, the risk of chronic kidney disease increased significantly with increasing triglyceride lipid profiles. This finding is supported by a study performed in Olsztyn, Poland [58], Taiwan [68], a review report in Ethiopia [59], and Dessie Referral Hospital, Ethiopia [69]. This could be because high triglyceride levels increase hypertension-related kidney damage by causing endothelial dysfunction, atherosclerosis, which impair renal blood flow and promote ischemic injury. These effects are mediated in part by lipotoxicity and inflammatory cytokine release, which further compromise vascular integrity within the kidney and impair kidney function. Additionally, excessive triglyceride accumulation in kidney cells damages podocytes, which are important cells in the filtration barrier of the kidney, leading to proteinuria, and subsequent tubulointerstitial fibrosis. As fibrosis progresses, kidney function progressively diminishes, thereby accelerating the transition to advanced chronic kidney disease [70,71].

The present study revealed that the risk of hypertensive chronic kidney disease was significantly increased by uncontrolled blood pressure. This finding is corroborated by other studies conducted in Germany [72], the Seattle USA [73], Kenya [74], Boma, the Congo [75], Tigray, Ethiopia [62], Jimma, Ethiopia [63]. This could be due to uncontrolled blood pressure increasing the pressure inside the glomeruli, causing glomerular hyperfiltration. This eventually results in proteinuria, glomerular hypertrophy, and nephron loss. Furthermore, persistently elevated blood pressure causes arteriosclerosis, which lowers blood flow to the kidneys and ultimately exacerbates the development of chronic kidney disease through renal inflammation driven pathways, including RAAS activation, oxidative stress, and infiltration of macrophages into renal tissue [76,77].

### Limitations

As a strength, a large sample size, and robust statistical methods were used. On the other hand, regarding the limitation of this study, behavioural and lifestyle factors, such as exercise, alcohol consumption, smoking, dietary approach, and stress, were not recorded on the patient’s medical cards, so this study did not incorporate these variables because of the retrospective cohort study design. As a result, further prospective follow-up studies are needed to incorporate these missing variables.

## Conclusion and recommendation

Our study revealed that hypertensive patients progressed to chronic kidney disease more rapidly as compared to other studies, which revealed the critical window period for intervention during the at-risk stage in Ethiopia. Moreover, the incidence of hypertensive chronic kidney disease unexpectedly increased in rural and semi urban districts of Ethiopia. This high burden challenges the implementation of SDGs to reduce premature mortality from non-communicable diseases, including CKD, by one-third. Thus, the high burden of CKD and rapid decline in kidney function among individuals with hypertension could be explained by obesity, uncontrolled blood pressure, and high triglyceride levels. This implied that uncontrolled blood pressure, obesity, and dyslipidaemia can be used as high-risk indicators for CKD among hypertensive patients. These findings from Ethiopia highlight a critical window for intervention during the at-risk stage, before CKD stage one state. This may be applicable to other low-resource settings in Sub-Saharan Africa, which share common challenges including a high burden of hypertension, late patient presentation, limited health care resources, and genetic predisposition to certain types of kidney disease. So, the results of this study could be externally generalized.

Thus, hypertensive individuals with obesity, uncontrolled blood pressure, and elevated triglyceride level should receive early intervention to prevent the early development of hypertensive kidney disease. Furthermore, routine screening of triglyceride levels should be advocated to initiate early management and prevent the progression of hypertensive chronic kidney disease. Health facilities should revise their medical record templates to include dedicated fields for key behavioural and life style variables, specifically physical exercise, alcohol consumption, smoking status, dietary habits, and stress levels, as well as strengthen documentation practices to capture this information routinely. Finally, we recommend community-based lifestyle modifications that target obesity reduction and improve blood pressure control.

## Supporting information

S1 File CKD Data Sets in excel format. (XLS)

## Abbreviations

AHR: Adjusted Hazard Ratio
BMI: Body Mass Index
CKD: Chronic Kidney Disease
CI: Confidence Interval
DALY: Disability Adjusted Life Years
DTUIRC: Debre Tabor University Institutional Review Committee
ESRD: End-Stage Renal Disease
FMOH: Federal Ministry of Health
GBD: Global Burden of Disease
GFR: Glomerular Filtration Rate
ICD-10: International Classification of Disease Tenth Revision
MCAR: Missing Completely at Random
NCD: Non-Communicable Disease
PHC: Primary Health Care
SDG: Sustainable Development Goal
WHO: World Health Organization
