# Supplementary material for: Chronic kidney disease progression rate and predictors in hypertensive patients: A 5-year retrospective follow-up study in Ethiopia
Source: PLoS One. 2026 Aug 13;21(8):e0355483. doi: 10.1371/journal.pone.0355483 (PMC13472377; doi:10.1371/journal.pone.0355483)
Supplement: Supplementary file 1 [file pone.0355483.s004.pdf]

Response to Comments

Article ID: PONE-D-26-04990

Article title: Chronic Kidney Disease Progression rate and predictors in Hypertensive Patients: A 5-Year Retrospective Follow-up Study in Ethiopia Reveals a Critical Window for Intervention

Dear Editors and reviewers,

We thank the editors and reviewers for your constructive feedback, which has significantly improved our manuscript. All comments have been carefully addressed in the revision. Below, we provide a point-by-point response to each comment. In addition, two files have been uploaded: a revised manuscript with track changes and a clean copy without track changes

Editor's comments and our response

Journal Requirements: When submitting your revision, we need you to address these additional requirements.

1. Please ensure that your manuscript meets PLOS ONE's style requirements, including those for file naming. The PLOS ONE style templates can be found at

https://journals.plos.org/plosone/s/file?id=wjVg/PLOSOne_formatting_sample_main_body.pdf and https://journals.plos.org/plosone/s/file?id=ba62/PLOSOne_formatting_sample_title_authors_affiliations.pdf

Response: Based on the comment we have made amendments of the manuscript to be in line with the PLOSE One style templates.

2. We note that one of the authors of the present submission (Marelign Tilahun) is listed as a member of the ethics committee on the approval document provided. Could you please explain what your ethical committee's policies are when an Author is also a member of the committee, and how this was handled in this specific case? Thank you very much for your response to this request.

Response: Thank you for your careful review and for raising this important ethical consideration. We acknowledge that the ethical approval letter for this study was signed by author Marelign Tilahun in his capacity as Corporate Director for Research, and Community Service at Debre Tabor University. Debre Tabor University's ethics committee policy states that any committee member who is also an author on a submitted protocol must recuse themselves from all discussions, deliberation, and voting. However, as the Corporate Director for Research and Community Service Directorate office, Dr. Marelign Tilahun has an administrative responsibility to sign approval letters as the head of the directorate. This is a procedural role, not an indication of active participation in the ethical review of this specific study. Dr. Marelign Tilahun was not involved in the peer review, discussion, or decision-making process regarding this study's ethical approval. The ethical review was conducted by independent members of the ethics committee who were not affiliated with the research team. Dr. Marelign Tilahun's signature on the approval letter reflects his ex-officio administrative duty to issue final decisions on behalf of the directorate, not personal endorsement or influence over the review outcome.

3. In the online submission form you indicate that your data is not available for proprietary reasons and have provided a contact point for accessing this data. Please note that your current contact point is a co-author on this manuscript. According to our Data Policy, the contact point must not be an author on the manuscript and must be an institutional contact, ideally not an individual. Please revise your data statement to a non-author institutional point of contact, such as a data access or ethics committee, and send this to us via return email. Please also include contact information for the third party organization, and please include the full citation of where the data can be found.

Response: We appreciate your concern regarding the data statement revision, particularly the data policy and the contact point. We have revised the data statement both in the online submission system and in the manuscript **(line number 493-495)** to reflect that we plan to share the datasets used in the current study as supplementary files accompanying this manuscript in the online submission system. Therefore, no external contact point is actually needed for data sets access.

4. When completing the data availability statement of the submission form, you indicated that you will make your data available on acceptance. We strongly recommend all authors decide

on a data sharing plan before acceptance, as the process can be lengthy and hold up publication timelines. Please note that, though access restrictions are acceptable now, your entire data will need to be made freely accessible if your manuscript is accepted for publication. This policy applies to all data except where public deposition would breach compliance with the protocol approved by your research ethics board. If you are unable to adhere to our open data policy, please kindly revise your statement to explain your reasoning and we will seek the editor's input on an exemption. Please be assured that, once you have provided your new statement, the assessment of your exemption will not hold up the peer review process.

Response: Thank you for your helpful comments regarding our data availability statement. We confirm that we are able and willing to adhere to PLOS ONE's open data policy. The datasets used in this study will be made freely accessible upon acceptance of the manuscript, with no restrictions. To avoid any delay in publication, we have already prepared our data sharing plan. Specifically, the data will be uploaded as supplementary files accompanying the manuscript in the online submission system. In addition, we have revised the data availability statement in both the manuscript and the submission form to reflect that the data will be freely available **(line number 493-495).**

5. Your ethics statement should only appear in the Methods section of your manuscript. If your ethics statement is written in any section besides the Methods, please move it to the Methods section and delete it from any other section. Please ensure that your ethics statement is included in your manuscript, as the ethics statement entered into the online submission form will not be published alongside your manuscript.

Response: Thank you for this reminder. Based on the request, we have moved the ethics statement to the Methods section of the manuscript and removed it from any other sections (**line number 267-273)**

6. If the reviewer comments include a recommendation to cite specific previously published works, please review and evaluate these publications to determine whether they are relevant and should be cited. There is no requirement to cite these works unless the editor has indicated otherwise.

Response: Thank you for the citation suggestions. We did not receive any recommendations from the reviewers regarding previously published works or self-citation. Based on the editor's concern, we have reviewed the recommended publications and determined that, one reference (https://doi.org/10.2147/JMDH.S361061) may not be directly relevant to the specific focus of our study, though it is related to the method of analysis. However, the other reference (https://doaj.org/article/c84ae8f6b3b242c59cc936b2f767f298) was considered conceptually relevant by the authors and has been included in the literature review for tool preparation. Therefore, we have not included the first reference in the revised manuscript, while the second remains included. We will, of course, follow any guidance from the editor on this matter, and we are happy to accept the Editors decision and revise it accordingly.

Reviewers' comments and author response

Reviewer #1 comments and author responses:

1. Dear Authors, the paper is interesting. Introduction should be enriched with epidemiological data. Material and methods are clear. Female population is almost twice that male. Please clarify this point.

Response: Thank you for your positive feedback and constructive suggestions. We have revised the Introduction section according to the comments by incorporating additional epidemiological data **(Line number 70-88).** Regarding the point that the female population is almost twice that of the male population, we have indicated this demographic data in introduction section (**line number 62).** In addition we have added a clarification for this point based on our data in the Results section **(line 283-285**) as Female participants (307, 60.2%) were in higher proportion compared with males (203, 39.8%), reflects the health care seeking behavior in this setting, where women are more likely to attend regular follow-up visits for chronic conditions including hypertension care.

2. Results are clear. Discussion needs to be empowered with pathophysiological arguments to strengthen and explain the findings. The role of inflammations could be displayed.

Response: Thank you for your positive feedback on the clarity of our results and for this constructive suggestion. We agree that incorporating pathophysiological arguments, particularly the role of inflammation, will strengthen the Discussion section. Accordingly, we have revised the discussion section by incorporating the role of inflammation in the pathophysiology process **(line numbers 413-420, 426-430, 440-443).**

3. Is the manuscript presented in an intelligible fashion and written in standard English? No

Response: We thank the reviewer for this honest and helpful assessment. We acknowledge that the manuscript requires significant improvement in language clarity and overall readability. In response, we have thoroughly revised the manuscript accordingly.

Reviewer #2 comments and author responses:

Thank you for sending along the manuscript, which is tackling the most relevant area in chronic disease management. To improve the clarity of some sections of the manuscript, Kindly attend to the following attached few comments made.

Response: Thank you for your positive and constructive feedback. We have carefully reviewed the attached comments and have revised the manuscript accordingly. A point-by-point response is provided below.

Comments

Title; Rapid Progression to Chronic Kidney Disease in Hypertensive Patients: A 5-Year Retrospective Follow-up Study in Ethiopia Reveals a Critical Window for Intervention

1. The topic is biased, kindly make the topic neutral. Rather rephrase as: Chronic Kidney Disease progression rate and predictors in Hypertensive Patients: A 5-Year Retrospective Follow-up Study

Response: Thank you for your insightful comment. We have revised the title as suggested to be more objective and reflective of the study's actual scope.

Abstract

2. Methods: What was the sampling strategy used to select the 510 records?

Response: We thank the reviewer for this important clarification. The sampling strategy used to select the 510 records was simple random sampling. Specifically, from the total available patient records meeting the initial inclusion criteria during the study period, we prepared a sampling frame based on their card numbers. We then used a random number generator to select the final 510 records. This approach minimized selection bias and ensured that every eligible record had an equal and independent chance of being included in the analysis. We have now stated this in the abstract's Methods section in short **(line number 36).**

Introduction

3. Chronic kidney disease (CKD) is a condition characterized by a progressive decrease in kidney function over time that results in decreased kidney capacity to excrete water-soluble waste products and adjust water, electrolytes, and acid-base balance. It affects millions of adults, particularly those with diabetes and hypertension. Kindly reference these claims

Response: Thank you for this valuable suggestion. We have added references to the suggested paragraphs and cited these paragraphs accordingly **(line number 57 &58**).

4. Chronic kidney disease is becoming increasingly common worldwide, and it is one of the most prevalent non-communicable diseases. Although there are many different and complex causes of CKD, its early signs are frequently subtle and rare. Kindly reference these claims

Response: Thank you for this important comment. We have revised the relevant paragraph in the introduction and added citations to support these claims **(line number 62 &63).**

5. In this section, literature focused more on prevalence (Existing cases of CKD) rather than the incidence of CKD (new cases), progression rate and predictors. There is need to cover what is known in terms of these 3 concepts, with indications of the exact gap in the context of the study.

Response: Thank you for this important observation. We have substantially revised the introduction section to include available evidence on CKD incidence, progression rates, and established predictors **(line number 70-88).**

Materials and methods

6. Cover the study design and period first, then study setting follows

Response: We have rearranged based on the comment **(line number 117&127)**

7. Study setting: Although the context of study setting is described, it is not clear which exact settings were used out of the 10? Is it that all the 10 hospitals were used as study setting or some were selected to be used and if yes, how was the selection done?

Response: Thank you for your comment. We have clarified this point in the revised manuscript: only three of the ten hospitals were selected as the study setting. The selection was done using a

lottery method (simple random sampling technique). The revised setting section now specifies this and names the three hospitals **(line number 135-137).**

8. Study design and period; We carried out an institution-based retrospective follow-up study to determine the progression time for the occurrence of chronic kidney disease and its determinants among hypertension patients who got follow-up care in selected hospitals between September 2019 and June 2024. The narrative have changed from predictors to determinants, which is which? Again, what about incident of CKD?

Response: Thank you for your careful observation. We have revised these paragraphs based on your comment, and we have made the manuscript consistent with your suggestions **(line number 118-120)**

9. Sample size determination and sampling technique: Three of the 10 hospitals in the North-Western Ethiopia, South Gondar zone district that provide hypertension care services were chosen for this study via a simple random selection procedure. This statement should appear in the description of the setting. Check my comment above

Response: Thank you for this important comment, we have revised the study setting by taking these sentences based on your comment **(line numbers 135-137).**

10. Dependent variable: Progression time to hypertensive chronic kidney disease. What about the incidence of CKD as the dependent variable?

Response: Thank you for this insightful suggestion. Based on your comment, we have now amended the manuscript to include both progression time to hypertensive CKD and incidence of hypertensive CKD as outcome (dependent) variables **(line number 168***)*. Our primary dependent variable was progression time to hypertensive CKD, as we used survival analysis (time-to-event data). However, we have also included incidence of hypertensive CKD as an additional outcome variable to indicate the occurrence of new CKD cases during the follow-up.

11. Limitations: On the other hand, regarding the limitation of this study, behavioural and lifestyle factors, such as exercise, alcohol consumption, smoking, dietary approach, and stress, were not recorded on the patient's medical cards, so this study did not incorporate these variables because of the retrospective cohort study design. The following limitation was given and these

are important predictors of CKD. Of course, a recommendation on further prospective follow-up studies to incorporate these missing variables was given and this can be a long term one. In the conclusion and recommendation, kindly indicate the recommendation in terms of revising the medical cards so that they capture the information and improving documentation in practice.

Response: Thank you for this valuable and practical suggestion. We agree that behavioral and lifestyle factors are important predictors of CKD. In response to your comment, we have now revised the Conclusion and Recommendation section to include a specific recommendation regarding medical record documentation. The revised text has been updated accordingly in the manuscript **(line numbers 469-473).**
