# Supplementary material for: Chronic kidney disease progression rate and predictors in hypertensive patients: A 5-year retrospective follow-up study in Ethiopia
Source: PLoS One. 2026 Aug 13;21(8):e0355483. doi: 10.1371/journal.pone.0355483 (PMC13472377; doi:10.1371/journal.pone.0355483)
Supplement: Supplementary file 2 [file pone.0355483.s002.odt]

Comments 
Rapid Progression to Chronic Kidney Disease in Hypertensive Patients: A 5-Year Retrospective Follow-up Study in Ethiopia Reveals a Critical Window for Intervention
The topic is biased, kindly make the topic neutral. Rather rephrase as: Chronic Kidney Disease progression rate and predictors in Hypertensive Patients: A 5-Year Retrospective Follow-up Study
Abstract
Methods: What was the sampling strategy used to select the 510 records?
Introduction
Chronic kidney disease (CKD) is a condition characterized by a progressive decrease in kidney function over time that results in decreased kidney capacity to excrete water-soluble waste products and adjust water, electrolytes, and acid-base balance. It affects millions of adults, particularly those with diabetes and hypertension. Kindly reference these claims
Chronic kidney disease is becoming increasingly common worldwide, and it is one of the most prevalent non-communicable diseases. Although there are many different and complex causes of CKD, its early signs are frequently subtle and rare. Kindly reference these claims
In this section, literature focused more on prevalence (Existing cases of CKD) rather than the incidence of CKD (new cases), progression rate and predictors. There is need to cover what is known in terms of these 3 concepts, with indications of the exact gap in the context of the study. 
Materials and methods
Cover the study design and period first, then study setting follows
Study setting: Although the context of study setting is described, it is not clear which exact settings were used out of the 10? Is it that all the 10 hospitals were used as study setting or some were selected to be used and if yes, how was the selection done? 
Study design and period; We carried out an institution-based retrospective follow-up study to determine the progression time for the occurrence of chronic kidney disease and its determinants among hypertension patients who got follow-up care in selected hospitals between September 2019 and June 2024. The narrative have changed from predictors to determinants, which is which? Again, what about incident of CKD? 
Sample size determination and sampling technique: Three of the 10 hospitals in the North-Western Ethiopia, South Gondar zone district that provide hypertension care services were chosen for this study via a simple random selection procedure. This statement should appear in the description of the setting. Check my comment above
Dependent variable: Progression time to hypertensive chronic kidney disease. What about the incidence of CKD as the dependant variable? 
Limitations: On the other hand, regarding the limitation of this study, behavioural and lifestyle factors, such as exercise, alcohol consumption, smoking, dietary approach, and stress, were not recorded on the patient’s medical cards, so this study did not incorporate these variables because of the retrospective cohort study design. The following limitation was given and these are important predictors of CKD. Of course, a recommendation on further prospective follow-up studies to incorporate these missing variables was given and this can be a long term one. In the conclusion and recommendation, kindly indicate the recommendation in terms of revising the medical cards so that they capture the information and improving documentation in practice.
